# How to assess experienced quality of care in nursing homes from the client’s perspective: results of a qualitative study

**DOI:** 10.1186/s12877-020-1466-7

**Published:** 2020-02-17

**Authors:** Katya Y. J. Sion, Hilde Verbeek, Bram de Boer, Sandra M. G. Zwakhalen, Gaby Odekerken-Schröder, Jos M. G. A. Schols, Jan P. H. Hamers

**Affiliations:** 1 Department of Health Services Research, CAPHRI Care and Public Health Research Institute, Faculty of Health Medicine and Life Sciences, Maastricht University, Duboisdomein 30, 6229 GT Maastricht, The Netherlands; 2 Department of Marketing and Supply Chain Management, SBE School of Business and Economics, Maastricht University, Tongersestraat 53, 6221 LM Maastricht, The Netherlands

**Keywords:** Quality of care measurement, Long-term care, Caring relationships, Relationship-centered care, World café, Focus group, Client perspective

## Abstract

**Background:**

The culture shift in nursing homes from task-oriented to person-centered care has created a need to assess clients’ experienced quality of care (QoC), as this corresponds best with what matters to them. This study aimed to gain insight into how to assess experienced QoC in nursing homes from the client’s perspective.

**Method:**

A qualitative study was performed consisting of a focus group with client representatives (*n* = 10), a focus group with nursing home staff (*n* = 9) and a world café with client representatives and staff recruited from the Living Lab in Ageing & Long-Term Care (*n* = 24). Three questions about assessing experienced QoC from the client’s perspective were addressed during data collection: 1) What content needs to be assessed? 2) What assessment procedures are needed? and, 3) Who needs to be involved in the assessment? Semi-structured questions, photo elicitation and creative writing were used to answer these questions. Conventional content analysis was used to analyze the data.

**Results:**

Participants indicated that experienced QoC mostly occurs within the interactions between clients, family and staff, highlighting the impact of relationships. They suggested assessments should focus on three aspects: 1) knowledge about the client, 2) a responsive approach, and 3) a caring environment. These can be assessed by having conversations with clients, their families and staff, and additionally observing the clients in their living environments. Sufficient time and resources are prerequisites for this. Additionally, the person performing the quality assessments needs to possess certain communication and empathy skills.

**Conclusion:**

It is important to include the perspectives of the client, family and staff when assessing experienced QoC, in line with the principles underlying relationship-centered care. In order to be feasible, it is recommended to incorporate quality assessments into the nursing homes’ daily routines. Further research with clients, family and staff in nursing homes is needed to develop a feasible, reliable and valid method that assesses experienced QoC from the client’s perspective.

## Background

Currently, Western countries are struggling to consistently improve quality of care (QoC) in nursing homes [1]. Reasons for this are changing expectations of what nursing homes should offer, an increase in the aging population, and high staff shortages and turnover [2, 3]. Many definitions of QoC exist and most relate to the Institute of Medicine’s criteria stating that care needs to be safe, effective, patient-centered, timely, efficient and equitable [4–6]. However, there has been a culture change from task-oriented to person-centered care, putting clients’ needs, wants, preferences and relationships more centrally in care provision in order to achieve high QoC in nursing homes [7–10]. Consequently, it has become more important to include the client’s perspective when assessing QoC and focus on what matters most to clients, i.e. the client’s experienced QoC [11]. Research has shown that clients’ and families’ experiences offer less tangible information on QoC, such as the importance of feeling at home, being empowered and maintaining dignity [12, 13]. These insights have resulted in the need to incorporate these perspectives when assessing experienced QoC in nursing homes [11, 14–16]. In the Netherlands, nursing home clients can live in three types of wards: somatic for those with physical deteriorations; psychogeriatric for those with cognitive impairment; and rehabilitations for those who are recovering from temporary physical impairment [17]. In 2016, the Dutch government introduced an updated policy on how to maintain and improve QoC in nursing homes [18]. This policy focusses on person-centered care and relationships, well-being, safety and learning from each other. In other countries similar developments are occurring [19].

The Individually Experienced Quality of Post-Acute and Long-Term Care (INDEXQUAL) framework presents experienced QoC from the client’s perspective as a process, consisting of a before (expectations), during (experiences) and after (assessment) phase within a certain context [20]. It acknowledges that care experiences occur mostly within interactions between the client, family and staff, in line with the principles of relationship-centered care and defines experienced QoC as the sum of perceived care services, perceived health outcomes and satisfaction. Many instruments have been identified that assess QoC in nursing homes [21]. However, research on experienced QoC has mainly focused on satisfaction, which is defined as the subjective evaluation of the gap between a health care recipient’s expectations and experiences with care [22, 23]. Other instruments address perceived health outcomes, which assess the client’s views on his or her health status [24].

Currently, there is growing interest to assess perceived care services, focused on relationships and practical issues, assessed with patient-reported experience measures [24]. A majority of these instruments are quantitative and give a rating on specific pre-defined topics, lacking information that explains *why* a certain rating is given and what can be done to improve it [21, 24]. These questionnaires limit the opportunity for respondents to divert beyond their pre-defined topics and address what may actually be of even more value to them. Whilst the results are useful for transparency and accountability purposes, there is a growing need to also monitor and improve the client’s individually experienced QoC [22, 25]. In line with these developments, qualitative approaches to assess experienced QoC are being developed and used more frequently. However, a majority of these instruments have not been developed according to the steps in the development and evaluation of a measurement instrument, starting with clearly defining the construct [26]. This has resulted in them also not having been sufficiently tested regarding their validity, reliability, ability to contribute to quality improvements and user-friendliness [21, 26]. Therefore, the aim of this study was to discover how to assess experienced QoC in nursing homes from the client’s perspective according to client representatives’ and nursing home staffs’ views. These insights will support the future development of a method to assess experienced QoC in nursing homes from the client’s perspective.

## Methods

### Study design

This was a qualitative study consisting of two focus groups and a world café. A focus group is a specific type of group interview in which group interaction is an explicit part of the method and participants’ thoughts can be explored [27]. The world café method is a specific type of group conversation in which a mix of participants share their knowledge and build further on each other’s ideas [28].

### Participants

For the first focus group, policy officers and nurses employed in a nursing home organization were invited to represent the nursing home staff’s perspective (hereafter referred to as staff). For the second focus group, client council representatives were invited to represent the voice of the clients (hereafter referred to as client representatives). Both focus groups consisted of homogenous groups to create a comfortable and safe environment for discussions [27]. For the world café, heterogeneous groups were formed to enhance the discussions and give participants the opportunity to learn from each other and create new ideas together [27, 28]. Policy officers, formal caregivers (such as nurses or physiotherapists), family, and client council representatives were invited to participate (hereafter referred to as world café participants). The difference between family and client council representatives is that family represent one client’s voice, whereas client council representatives have a position within the nursing home to represent the voice of all clients without having to be directly connected to one specific client. This study planned to include clients living in nursing homes as well; however this was considered challenging as many clients in nursing homes suffer from cognitive decline [17]. After having performed two pilot interviews with clients living in somatic wards, without cognitive impairment, it became apparent that this was not feasible. Whilst clients were able to talk about how they perceived the care they received they were not able to distinguish this from how they believed this should be assessed.

Whilst purposive sampling was used to select the main groups of participants directly involved in nursing homes; convenience sampling was used to select the participants within these groups. Staff engaged with QoC policy assurance were selected as they were considered most knowledgeable about the developments in the nursing home setting, and client representatives were selected as they were closely involved with clients and considered knowledgeable about what is important to clients. Participants were recruited from seven nursing home organizations within the Living Lab in Ageing & Long-Term Care South Limburg (the Netherlands), via an information letter providing information about the aim of the study, a description of the participants, the location and date, confidentiality and how to participate [29]. The information letters were distributed by the contact persons within the organizations. Participants could register by informing the contact person or the lead researcher of the study by phone or e-mail. For each focus group the aim was to include 8 to 12 participants [30], and for the world café the aim was to include 20 to 28 participants [28]. All participants provided written informed consent and could sign up for a newsletter to stay informed on the results of the research.

### Data collection

Data collection took place between May and July 2017 at the university. The focus group with staff was performed first to position the need for a new method of assessing experienced QoC. This was followed by the world café in which participants could brainstorm, share ideas and discuss together. The focus group with client representatives was performed last, in order to gain more in-depth knowledge about the clients’ needs. The research team established data saturation was reached after the last focus group [31].

All discussions were focused on the *content* to assess, the *procedure* of the assessment and *who* to involve during the assessment. Table 1 shows the main characteristics and interview guide for each group discussion. The interview guide was specifically developed for this study. All participants were asked to complete a brief questionnaire on their age, gender and professional background.

**Table 1:** Overview of data collection methods

	Data collection	Question(s)	Duration / Researchers
Focus group staff	Semi-structured questions	1. Without any restrictions, how would you assess how clients experience the quality of care they receive in nursing homes? 2. Which topics need to be discussed during the quality assessment? 3. What assessment procedures are needed? 4. Who needs to be involved in the assessment?	1 h / Health Scientist (first author) and Associate Professor in Long-Term Care Design
Focus group client representatives	Photo elicitation	1. Please select an image that represents how quality of care in nursing homes should be assessed from the client’s perspective?	1 h / Health Scientist (first author) and Professor in Care of Older Persons
World café	Photo elicitation Post-its and writing material	1. Please select an image which represents your expectations of care in a nursing home from the client’s perspective? 2. Please select an image which represents your experiences of care in a nursing home from the client’s perspective? 3. Please select an image which represents how quality of care in nursing homes should be assessed from the client’s perspective? 4. Who is involved in a client’s network?	2,5 h / Health Scientist (first author) and Associate Professor in Long-Term Care Design and 4 researchers in aging (moderators).

#### Focus groups

The one-hour focus group with staff was guided by semi-structured questions; as they were considered to already have thoughts on the topic. The one-hour focus group with client representatives used photo elicitation in order to trigger discussions [32]. As the research question was considered quite broad, images were used to support participants to structure their thoughts [33]. Photo elicitation can stimulate a deeper layer of a person’s consciousness and unveil participants’ underlying views and beliefs [32]. This study used the My Home Life Scotland© image pack consisting of approximately 100 different images, varying from two people holding hands, to an image of puzzle pieces [34]. The focus group started by inviting client representatives to select an image that best captured how they felt experienced QoC in nursing homes should be measured. Hereafter, participants explained why they chose a specific image and this was followed up by in-depth questions facilitating further discussion. Both focus groups were led by one researcher and supported by another researcher from the research team. Discussions were audio recorded and field notes were taken. Preliminary results were presented to both groups for interpretation and discussion.

#### World café

The world café method covered four themes, each focusing on a specific question (Table 1). Questions 1, 2 and 3 used photo elicitation with the My Home Life Scotland© images to stimulate discussion. Question 4 used post-its and colored pens to create an overview of all stakeholders in a client’s network. First, participants were informed about the definition of experienced QoC in nursing homes from the client’s perspective, to assure discussions would focus on personal experiences and not on standardized quantitative outcomes such as the prevalence of pressure ulcers or malnutrition. Second, participants were invited to take a random seat at one of the four tables representing a question. In three consecutive 30-min rounds, separate groups consisting of 4 to 8 participants were encouraged to discuss the question. After each round, participants swapped seats and continued a discussion about another theme at a different table. A moderator remained seated at the table to introduce the new theme and explain what the previous group had discussed [28]. The moderators had experience in guiding groups and world cafés, and received a 1-h training. During this training the lead researcher provided information on the aim of the world café, and how to stimulate and capture discussions. Additionally, moderators were assigned to their research question and were provided with the opportunity to ask questions. Discussions were written down in keywords on sheets of paper covering the tables, and subsequently summarized. Participants started each round by writing down their thoughts on post-its and laying these onto the table sheet. After the three sessions, there was a plenary session in which each group presented the results of the specific theme, and field notes were taken by the researcher. All moderators provided the lead researcher with a summary of the three rounds including explanations for each of the chosen images for the questions using elicitation. After interpreting these summaries, the lead researcher had conversations with all moderators to confirm that the interpretations of the results were correct.

### Data-analysis

Conventional content analysis was used to analyse the collected data [30, 35]. First, audio recordings from both focus groups were transcribed, and the extensive summaries and table sheets from the world café were prepared for analysis. Then, the first author familiarised with this data and gained a deeper understanding by reading all transcripts and summaries multiple times. Hereafter, the first author identified key thoughts and concepts by means of open coding. Concepts such as knowing the client, expectations, methods of assessing QoC, prerequisites for assessments, and perspectives were coded and a code tree emerged. A top-down approach was used to create overarching categories which were based on the main *content*, *procedure* and *who to involve* themes that guided data collection. A second researcher validated the code tree, by coding sections of the transcript with the same code tree. This was compared with the first author’s coding to identify similarities and differences. Differences were resolved with the research team and adjusted throughout the entire coding process. Data were analysed with MAXQDA version 18.0.3 software [36].

### Trustworthiness

Multiple actions were involved to enlarge the trustworthiness of this study [37–39]. Participants were invited from seven long-term care organizations in the region, which contributed to the credibility of this study. Method triangulation was apparent as two focus groups and a world café were performed with the same aim [40]. Data triangulation was apparent as participants with different roles in the nursing home setting participated [40]. Furthermore, the research team engaged in reflexivity acknowledging and discussing their views on QoC assessments and the impact of their views and backgrounds on the research process [40]. Data analysis was performed by two researchers, known as investigator triangulation [40]. In order to enhance dependability, the procedures followed in this study were described in detail, and to increase the confirmability, the main results were summarized at the end of both focus groups and the world café [39]. Participants were encouraged to further explain their thoughts, and correct or add information when necessary. Detailed descriptions of the findings have been supported with quotes from both focus groups and the world café, increasing the transferability of the presented findings in this study [38]. Additionally, a group of experts involved in national long-term care policy making was consulted after data collection to discuss and validate the findings.

### Ethics approval

The study protocol was approved by the medical ethics committee of Zuyderland (17-N-86). Information about the aim of the study and the expected burden of the focus group or world café session was provided to all participants in advance by e-mail. Participation was strictly voluntarily for all participants. Before the start of each gathering, written informed consent to contribute to the study was given by all participants. Participants were allowed to withdraw from the study at any moment. In order to guarantee privacy and anonymity of participants, no names or institutions were documented.

## Results

A total of 38 stakeholders participated in this study as presented in Table 2.

**Table 2:** Characteristics of participants

	Focus group staff (*n* = 10)	Focus group client representatives (*n* = 9)	World café (*n* = 24)
Gender % (n) female	100% (10)	33% (3)	92% (22)
Age years mean [min; max]	42 [27; 54]	71 [61; 83]	43 [22; 68] ^**a**^
Participants (n)^b^	Staff: • Policy officer ^**c**^ (8) • Formal caregivers (2) *Nurses (2)*	Client representatives: • Client council representatives (9)	Staff: • Policy officer ^**c**^ (7) • Formal caregivers (12) *Nurses (8)* *Physiotherapists (2)* *Occupational therapists (2)* Client representatives: • Family (3) • Client council representatives (2)

^**a**^ *n* = 23, data from one participant is missing

^**b**^ three policy officers and two client council representatives participated in both a focus group and the world café

^**c**^ policy officers were employed at a nursing home organization and were occupied with quality assurance within their organization

**Fig. 1:**
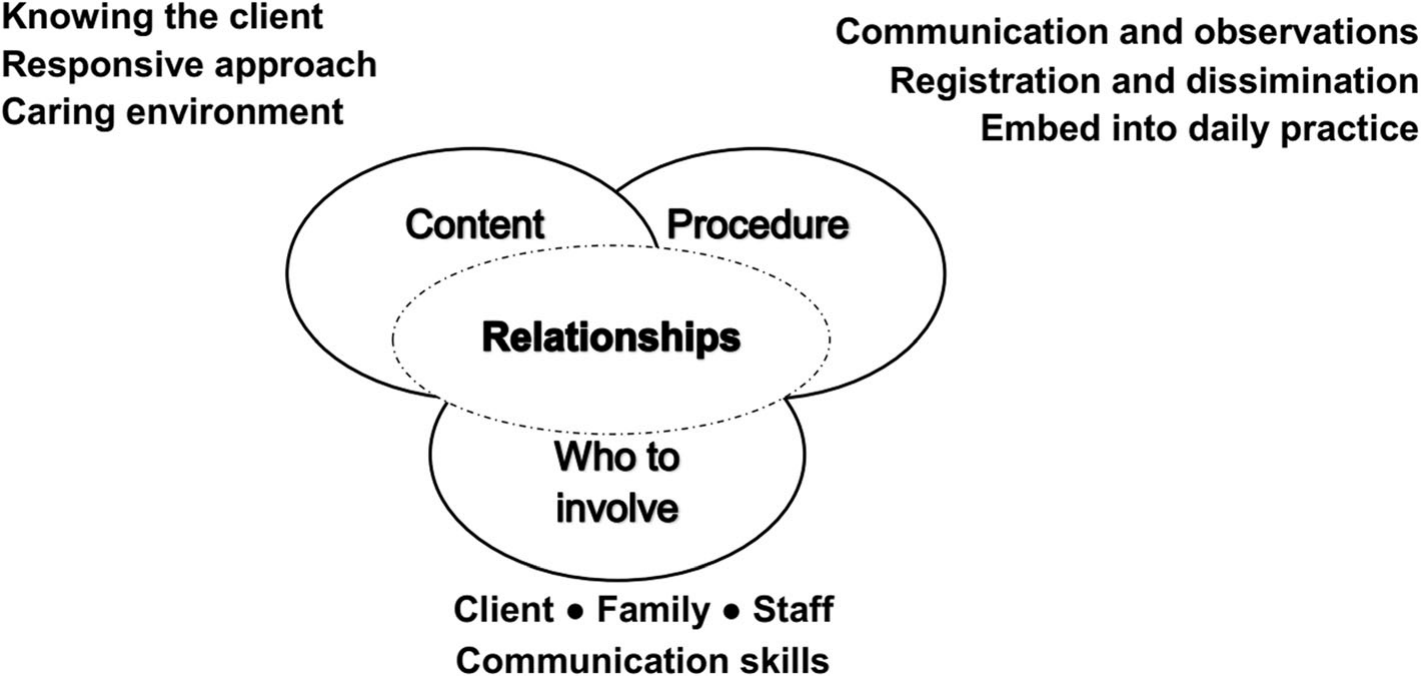
Identified topics from the focus groups and world café regarding how to assess experienced quality of care in nursing homes from the client’s perspective

Figure 1 provides an overview of the topics that were discussed by the participants. All participants emphasized the importance of relationships for care experiences and their assessments. They reflected that a great part of experienced QoC occurs within the interactions between the clients, family and staff. The following sections will present participants’ views on the content, procedure and who to involve, and the importance of relationships when assessing experienced QoC in nursing homes from the client’s perspective.

### Relationships

One overarching topic occurred in the content, procedure and who to involve sections: the importance of relationships. Participants in each group believed that taking time to establish a relationship with the client and show genuine interest is essential for meaningful conversations. It is important to explore and experience the client’s life together and adopt a tailored approach during these conversations. Staff viewed experienced QoC to be highly influenced by relationships between clients and their formal caregivers. Client representatives added family to this equation, as they are often involved in expressing the clients’ preferences and needs. Additionally, the relationship between the client and the person assessing experienced QoC can affect the outcome of QoC assessments. According to client representatives, true commitment, trust, empathy, openness, attention for what is being said, and a level of understanding are needed within relationships. Speaking the same language could contribute according to staff and world café participants. For example, speaking a specific dialect or approaching someone with the title or name they prefer. In conclusion, relationships were seen as the pillars of experiencing and assessing experienced QoC.

*“Quality of care is related to emotions and experiences in all phases of the disease. To be able to measure that, you must be able to experience and feel this, which requires a continuous professional relationship.”* (Client representative).

### Content of the quality assessment

Participants in all groups suggested assessments should focus on three aspects: 1) knowing the client, 2) adopting a personal approach for each client, and 3) creating a caring environment.

#### Knowing the client

Participants in all groups mentioned it is important to get to know the clients and their expectations, wishes and needs in order to make them feel at home. This already starts when a client has not moved to the nursing home yet, as this can contribute to a smooth move. For clients and their family it can be a big step to move to an unfamiliar place, which might feel threatening, and therefore prior to moving to the nursing home it was considered beneficial for the experience, to already know who the client is. Client representatives and world café participants added that it is important to know a client’s history, even though a client’s demands and expectations can shift and change during the disease process. Nursing homes are expected to know what clients and their families expect, and clients and families are expected to know what they can expect from the nursing home. Everyone’s norms and values differ, and therefore participants expressed the importance of tailored care. By discovering what a client finds pleasant, values will become visible and care can be tailored. Both staff and client representatives acknowledged the importance of relationships to achieve this.

#### Responsive approach

Participants mentioned that it is especially important when agreements have been made, that these are fulfilled within a reasonable timeframe. As experienced QoC was approached as a subjective concept, what to assess differs between clients, and therefore client representatives recommended to decide on this together with the client. Client representatives approached QoC as a personal experience related to less tangible concepts such as emotions and quality of life. They stated clients are seeking for closeness, affection, compassion, attention, and relationships, regardless of the severity of their physical and/or cognitive disabilities. Therefore, when assessing experienced QoC it is important to consider these aspects. It was suggested to assess if clients can organize their daily routines as they wish, and whether the nursing home is adhering to these wishes and fulfilling the client’s needs.

*“It’s in the small things. When a client calls that, he needs to go to the toilet for example. And the nurse replies [agitated] she’ll be right there. He does not feel taken seriously”* (Staff).

#### Caring environment

Participants discussed the importance of creating a safe and caring environment in which clients can rest, feel at home and feel secure. World café participants explained that a safe environment consists of more than alarm systems and locks, but actually touches upon the feeling of being safe at “home”. Staff mentioned there are countless possibilities to make someone feel more at home, however they also touched upon the fact that there is a certain limit, and sometimes nursing homes may not be able to meet the client’s expectations. This conflicting interest in wanting to provide to the individual’s needs, whilst simultaneously seeing countless limitations is a constant struggle. When assessing experienced QoC, it is important to acknowledge the client’s environment as well.

*“I think we [the nursing home] also need to stay honest. We try to match the home situation. We can decorate the home nicely with your [the client’s] own furniture and TV and photos and all, but it is no longer 100% like at home. I think you should always be realistic about that. We try to do everything as homely as possible and respect other’s values as much as possible. And yet there are certain limits.”* (Staff).

### Procedure of the quality assessment

Participants addressed the following aspects that need to be taken into account when assessing QoC from the client’s perspective: conversations and observations to measure, registration and dissemination of information, and embedding the assessment into practice with sufficient time and resources.

#### Conversations and observations

Participants in all groups clearly indicated that whilst they did not know in detail what the best procedure would be to assess experienced QoC, in their opinions existing standardized questionnaires do not sufficiently capture experienced QoC. Reasons for this were that they trigger socially desirable answers, lack the space to capture feelings, are considered too difficult, and focus too much on specific pre-defined topics. Staff emphasized the importance of the story behind a quantitative rating. Participants did mention numerous examples of possibly feasible methods to measure experienced QoC, however not providing details on what these procedures would exactly entail. The most frequently mentioned method was to have regular conversations addressing questions such as “What is important to the client?” or “What does the client expect from the nursing home?”. World café participants highlighted the importance of proper communication, especially between clients, family and staff. This requires actual sincerity during conversations, providing each other with time, space and attention. Additionally, they suggested a positive approach could support these conversations. Focus on what is going well and how to do more of this, and thinking in possibilities instead of limitations.

*“Have regular 10 minute conversations with the client, even when it seems there is nothing to discuss. Take a seat, sympathize and have a cup of coffee together [during daily care].”(*Client representative).

Participants indicated that not all clients might be capable of having conversations, because of their decline in health status and cognitive abilities. However, client representatives specifically stressed the importance of always trying to communicate with the client first. Observations were suggested to be of added value. Client representatives more specifically mentioned that facial expressions give away a lot of information, whereas world café participants focused more on participated observations in which the observer experiences the care environment. In line with observations, several world café participants highlighted the value of assessing QoC by combining speaking (i.e. conversations), hearing (i.e. listening), seeing (i.e. observing), smelling (i.e. cleanliness) and feeling (i.e. the atmosphere), which portrays a more complete picture of the actual daily experiences and interactions.

Both staff and client representatives mentioned the smiley method to roughly monitor how a situation is experienced, however acknowledging it is not sufficient to capture the full spectrum of experienced QoC. This method captures green (happy), orange (neutral) and red (unhappy) emotions. After an experience, the client or family member can evaluate by selecting the emotion that corresponds best to how they felt at that specific moment.

#### Registration and dissemination of collected information

Participants highlighted the importance that something is done with the information and that the client and family can see that (reciprocity); however, there was no agreement on how to achieve this. World café participants mentioned that a substantial amount of knowledge about the clients is present within the nursing home, however not registered and/or disseminated in a proper way. This could result in important knowledge about a client not reaching all caregivers. It was considered challenging to register information objectively and to the point. Staff suggested the use of grades from for example 1 to 10, however also immediately realized these do not provide information on what exactly is going well and what needs improving. Both a staff member and a client representative gave a similar example of the one page profile, in which a short list of essential client preferences and needs is portrayed in the client’s room.

*“Unfortunately, many promises are often made but few actions are undertaken.”* (Participant in world café at table topic 2).

Additionally, participants appeared to have different reasons to assess experienced QoC. Whilst staff highlighted the need for a proper balance of providing clients the space to tell what is important to them, and providing the nursing home constructive information that can be used to identify trends and improve the experienced QoC; client representatives aimed at assessing experienced QoC to improve the client’s individual care experience. These differences in aims support the complexity of how best to assess, register and disseminate experienced QoC information.

#### Embedding into daily practice

A majority of the participants recommended to assess continuously, as one assessment captures only a snapshot of reality, and therefore it was suggested to measure at multiple moments. Client representatives mentioned measurements should not be seen as big official moments. Whilst challenging, they recommended for measurements to have a low-threshold and be embedded into daily practice. Staff were more specifically discussing the need for a fixed frequency in the quality measurement, whilst keeping it feasible.

Participants indicated that nursing homes need to provide sufficient resources for quality measurements. Some considered the use of conversations and observations to be time consuming, whereas others noted that the conversations might be able to replace the content of the conversations that are already being held. Staff were searching for a balance between standardized checklists for benchmarking versus regular and tailor-made conversations.

*“Everything revolves around time. Time to be there, to listen, to take care of, to fill out forms. Time to let the client live his or her own life and if this becomes challenging, take time for that. Create time when needed. Time is also a precondition for staff.”* (Participant in world café at table topic 2).

### Who to involve in the quality assessment

Participants agreed it would be beneficial to include multiple perspectives in the quality measurement, to get a better overall view of experienced QoC. Most important, include the client, even when he or she might suffer from a cognitive decline. Whereas others also tend to have knowledge about the client, it was considered important to not surpass the client when measuring QoC from the client’s perspective. Clients are quite often still capable to express their wants and dislikes, and incorporating this perspective was considered crucial. Client representatives emphasized the importance of not making assumptions of what clients want or think, but to always ask them.

*“What strikes me is that people with dementia are often underestimated. They often can indicate what they like and don’t like… For example, people with dementia can also indicate: I want to go for a walk more often, I am just sitting inside and there is no one for me*.” (Staff).

Participants mentioned the family perspective can provide additional information about experienced QoC, however they do not always have the same views and preferences as the client. Participants indicated that when in doubt, preferences expressed by the client outweigh the family’s opinion. It was considered to be of added value to include the family’s own expectations and experiences, as these also influence the relationships and experienced QoC. Therefore, staff recommended to ask family what they think and feel, instead of asking them as a proxy on behalf of the client.

*“That is also a part of being attentive. Just asking a client or family member:’ how are you doing?”* (Staff).

Additionally participants mentioned that formal caregivers have plenty of knowledge about the client too. However, it is important they do not only reason from their medical background, but also from their knowledge of who the client is. World café participants mentioned formal caregivers, just like family, have their own expectations and experiences which can influence their assessment of experienced QoC.

*“Enter into conversations with different groups; the client, family members and caregivers.”* (Participant in world café at table topic 3).

Participants were not sure who needs to perform the quality measurement. On the one hand someone close to the client, because of the established relationship and the convenience of immediately solving problems. On the other hand, someone from outside might be better at objectively capturing experienced QoC, and allow clients to express themselves without being in a care dependent position. Dependency could result in clients and families not being completely open and honest, because they fear negative consequences for the client’s daily care. Participants did agree whoever performs the assessment needs to possess certain communicative skills and be motivated to get to the core. Staff and client representatives mentioned caregivers are doers, and therefore it is important to show them how to have these meaningful conversations and coach them on the job.

*“Family members often asked me [policy officer]: ‘Do you work for the nursing home organization? I don’t want dad or mum to be the victim of what I am saying’.”* (Staff).

## Discussion

The aim of this study was to discover the main needs regarding how to assess experienced QoC in nursing homes from the client’s perspective. The main findings related to the content, procedure and who to involve in the experienced QoC assessments, all implied that relationships form an important aspect of how care delivery is experienced and how it can be assessed. It became apparent that assessing experienced QoC is complex and no one has the perfect solution as to how this should be done. Participants provided pros and cons for most themes that were discussed. Results did show assessments should address if staff knows the client, responds to the client’s needs and has created a caring environment for the client, by having meaningful conversations with clients, their family and staff, as they are all part of the care experience. These conversations can be supported by observations and should be embedded into the existing care routines.

Findings in this study confirmed the importance of relationships when receiving and assessing care. Caring relationships have been defined as ‘human interactions grounded in caring processes, incorporating physical work (doing), interaction (being with), and relationship (knowing each other)’ [41]. Relationship-centered care emphasizes the necessity of caring relationships in order to achieve quality health care outcomes [42, 43]. This implies that care experiences occur during the interactions between the clients, family and staff, who all have their own ideas on what high QoC in nursing homes is [20].

This study confirms that what is assessed should reflect what matters most to the client [22]. The outcome of a client’s QoC assessment depends on whether the nursing home has met the client’s expectations and fulfilled his or her needs [44]. A recent meta-synthesis of older people’s experiences of care concluded a client’s main goal is to retain the meaning of being alive [13]. It is important to meet a client’s priorities; however there is a gap between a nursing home as a corporate culture and what clients perceive as good QoC [44]. Additionally, there is a gap in client and family quality ratings, as family is satisfied when the environment, staff and meals meet their standards [45–47]; whereas clients are satisfied when they feel at home and can retain their meaning of being alive [13, 44]. These differences confirm the importance of being cautious when family members assess quality as a proxy. They do not always know how the client feels and how services are being delivered [16, 46]. Therefore in order to increase the validity of quality results, it is essential that not only the client, but also family and staff are asked how they are experiencing the care process [44, 48, 49].

In order to identify the needs, feelings and experiences from the different perspectives, our findings suggest re-occurring meaningful conversations. Research has confirmed that standardized questionnaires are not sufficient to fully capture experienced QoC, and that qualitative data from conversations are very valuable to give care recipients a voice and get in-depth information on experienced QoC [22, 44, 50, 51]. Observations are considered of additional value to capture experienced QoC in nursing homes, as it can sometimes be challenging for clients to verbally express themselves [52]. This is however considered time-consuming and therefore sufficient time and resources are a prerequisite [48]. Additionally, it needs to be considered that clients and their families are dependent on staff, and may fear retribution when being completely honest about their experiences [53]. Therefore, it is important that the right person has conversations about experienced QoC. Whilst it remains unclear who this person should be, space needs to be created to form a trusting relationship, to be able to have meaningful conversations. This has been confirmed by others, who also perform research in the nursing home setting based on the relationship-centered care principles [54]. An advantage of having the formal caregiver perform the QoC conversations, is that they can immediately take action to improve QoC. These conversations could be incorporated in the daily care processes and the nursing home’s culture. In order to disseminate information, the content of daily work meetings could for example be changed. Instead of using these to discuss everyday processes, they could be used to discuss the client’s needs and wishes. In order for this to be successful, formal caregivers will need to improve how they reflect on the care provided and on their own competencies [55, 56]. It could be beneficial to adopt an appreciative inquiry approach, because whilst traditionally quality monitoring and improvements focus on identifying and solving problems, appreciative inquiry focusses on what is already working and how this can be done more frequently [57]. Adopting this positive approach has been proven to work motivating, encouraging and improve QoC in nursing homes [58, 59].

Furthermore, results confirmed that different groups have different reasons to assess experienced QoC [1]. Regulators want information for benchmarking purposes and local authorities use information for resource allocation decision-making. Whereas formal caregivers use quality information for internal quality improvement and learning from each other, clients and their family use quality information to select their providers, and to provide information about their experiences [1]. The output of quality results may differ depending on the purpose of the quality assessment, for example aggregated results on nursing home or organization level may be used for benchmarking, whereas individual or ward level results may be used for quality improvements. Therefore, it is important to define for what purpose experienced QoC is being assessed, prior to performing the assessment.

### Strengths and limitations

Some methodological considerations had to be made in this study. Clients in nursing homes were not directly participants during data collection. The set-up of this study, using many interactive and group discussions, may not have been a suitable method for clients living in nursing homes, due to their frailty and often cognitive impairments. We recommend future studies to adopt an inclusive approach by amending study designs to clients’ needs and capabilities. Research has shown that supportive approaches, such as visualization materials and simplified language can support the inclusion of this important population [60–62]. To assure the client’s voice was represented in the current study, client representatives were invited, as this is their main task within their position and they represent the voice of many more clients at the same time. They were considered to have a helicopter view of what issues are important to clients as they interact with a large variety of nursing home clients on a frequent basis.

An advantage of this study is that different methods were used to collect data, making it possible to personalize data collection to the needs of the stakeholders involved. Whilst it was expected that staff would be able to have meaningful discussions about the topics by means of supportive semi-structured questions; client representatives received visual stimuli to support them in answering the research question. For the heterogeneous group, the world café with supporting stimuli was used in order to create a comfortable environment with no visible hierarchy. A disadvantage of using different methods is that it was more challenging to compare and analyze the collected data, as this was collected with different questions and recorded with different resources such as audio and field notes. Whilst the world café method is an acknowledged research method, it is challenging to capture the findings without audio recordings in this deliberately created informal setting [28, 63]. To overcome this challenge, we used moderators that had sufficient knowledge on the topic, in order to assure they were capable of understanding and extensively summarizing the main findings.

Other studies have investigated which themes are considered important to client’s regarding their experienced QoC in nursing homes [13, 64, 65]. However, these studies mainly focused on *what* is important to clients, and not on *how* this needs to be assessed and *who* should be involved. To our knowledge, this is the first study that has combined different qualitative research methods and included client representatives’ and staffs’ views in the nursing home setting to find answers to these main questions.

## Conclusion

The findings of this study show that focusing on caring relationships is fundamental when assessing experienced QoC in nursing homes from the client’s perspective. In order to identify what really matters most to clients, there is a need for meaningful conversations with the client, family and staff about their experienced QoC and interactions with each other, supported by observations. Prerequisites for successful assessments are that the person performing these assessments need to possess certain communicative skills and the assessments should be embedded into daily practice, for example during the client’s yearly multidisciplinary consultation. Additionally, the results of the measurement need to be used to visibly improve the experienced QoC, as measuring needs to be done with a clear purpose. Adopting a positive, appreciative inquiry, culture could enhance nursing homes’ support, involvement and implementation of a new method to assess experienced QoC. The findings of this study can be used to develop a user-friendly, feasible, reliable and valid method that assesses experienced QoC from the client’s perspective. Further research should be performed in close collaboration with clients, their families and staff in nursing homes to ensure the developed method will meet everyone’s needs.

## Data Availability

The transcripts and summaries analyzed during the current study are not publicly available to guarantee anonymity and confidentiality for all participants, but are available in Dutch from the corresponding author on reasonable request.

## Abbreviations

QoC: Quality of Care
